# Achieving More with Less: A Lightweight Deep Learning Solution for Advanced Human Activity Recognition (HAR)

**DOI:** 10.3390/s24165436

**Published:** 2024-08-22

**Authors:** Sarab AlMuhaideb, Lama AlAbdulkarim, Deemah Mohammed AlShahrani, Hessah AlDhubaib, Dalal Emad AlSadoun

**Affiliations:** Department of Computer Science, College of Computer and Information Sciences, King Saud University, P.O. Box 266, Riyadh 11362, Saudi Arabia; 442200432@student.ksu.edu.sa (L.A.); 442200440@student.ksu.edu.sa (D.M.A.); 442201355@student.ksu.edu.sa (H.A.); 442201417@student.ksu.edu.sa (D.E.A.)

**Keywords:** human activity recognition (HAR), sensor data, deep learning, data augmentation, convolutional neural networks (CNNs), long short-term memory (LSTM), Inertial measurement unit, lightweight models

## Abstract

Human activity recognition (HAR) is a crucial task in various applications, including healthcare, fitness, and the military. Deep learning models have revolutionized HAR, however, their computational complexity, particularly those involving BiLSTMs, poses significant challenges for deployment on resource-constrained devices like smartphones. While BiLSTMs effectively capture long-term dependencies by processing inputs bidirectionally, their high parameter count and computational demands hinder practical applications in real-time HAR. This study investigates the approximation of the computationally intensive BiLSTM component in a HAR model by using a combination of alternative model components and data flipping augmentation. The proposed modifications to an existing hybrid model architecture replace the BiLSTM with standard and residual LSTM, along with convolutional networks, supplemented by data flipping augmentation to replicate the context awareness typically provided by BiLSTM networks. The results demonstrate that the residual LSTM (ResLSTM) model achieves superior performance while maintaining a lower computational complexity compared to the traditional BiLSTM model. Specifically, on the UCI-HAR dataset, the ResLSTM model attains an accuracy of 96.34% with 576,702 parameters, outperforming the BiLSTM model’s accuracy of 95.22% with 849,534 parameters. On the WISDM dataset, the ResLSTM achieves an accuracy of 97.20% with 192,238 parameters, compared to the BiLSTM’s 97.23% accuracy with 283,182 parameters, demonstrating a more efficient architecture with minimal performance trade-off. For the KU-HAR dataset, the ResLSTM model achieves an accuracy of 97.05% with 386,038 parameters, showing comparable performance to the BiLSTM model’s 98.63% accuracy with 569,462 parameters, but with significantly fewer parameters.

## 1. Introduction

Human activity recognition (HAR) is a transformative field with diverse applications in healthcare, fitness, military, and robotics. Caregivers can monitor older adults’ activities to detect the need for assistance [1], while physical therapists can provide real-time feedback to ensure patients perform exercises correctly [2]. In fitness and training scenarios, HAR can track movements, count steps, and calculate calorie expenditure to support overall wellness [2]. Additionally, HAR is employed in surveillance systems to detect threats and inform decision-making in critical infrastructure and combat situations [3]. In general, HAR aims to analyze and predict human behavior through activity signals collected from a variety of sensors such as magnetometers, gyroscopes, accelerometers, camera and LiDAR [4]. However, as smartphones have become ubiquitous, HAR models leveraging their inertial sensors (e.g., gyroscope, accelerometers) have gained traction, providing an unobtrusive solution for monitoring daily activities [4]. The research in this manuscript is focused only on HAR from mobile sensors including gyroscope (i.e., a sensor used for measuring the orientation and the angular velocity) and accelerometer (i.e., an electronic sensor for measuring the acceleration forces acting on an object). However, the computational complexity of traditional HAR models presents challenges for deployment on portable devices with limited resources.

Human-engineered feature characteristics are the fundamental components upon which shallow machine-learning models were constructed [5,6,7,8,9]. However, feature engineering is inherently time-consuming and subject to the influence of human biases and assumptions. To address this issue, researchers have explored the use of deep learning, which has revolutionized HAR with its automatic feature extraction capabilities [4]. Several relevant studies have investigated the methodologies of one-dimensional (1D) and two-dimensional (2D) convolutional neural networks (CNNs), as well as recurrent neural networks (RNNs). A 1D-CNN for HAR that employs a divide-and-conquer-based classifier with two stages was proposed by [10]. The first stage includes a binary classifier for recognizing abstract activities designated “dynamic” or “static”, while the second stage includes two multi-class 1D-CNN models for identifying individual activities for each of the binary classifications. The disadvantage is that each stage is dependent on the one before it, because if any mistakes are made at the start, the model will not be able to achieve correct action recognition. Dua et al. [11] proposed a multi-input CNN-GRU model for HAR comprising a three-head architecture and uses three different convolutional filter sizes to capture various spatio-temporal dependencies. Ragab et al. [12] introduced the random search 1D-CNN for HAR. Lee et al. [13] developed a deep learning model for semantic segmentation in HAR focusing on transition activities using a Multi-Channel CNN and an attention layer. An attention layer further refines feature focus, improving model performance on transition activity recognition. Zhang et al. [14] proposed a 1DCNN-ResBiLSTM-Attention model that combines 1D-CNN, residual bidirectional Long Short-term Memory (BiLSTM), and attention mechanism to improve the accuracy of recognizing similar activities by leveraging the distinctive leg movement patterns, achieving enhanced performance in activity recognition. Mehmood et al. [15] drew inspiration from DenseNet [16] and proposed an architecture that utilizes inertial sensors, with all previous feature maps available to all the layers ahead of it.

2D-CNN were employed for HAR [17,18,19]. Researchers [20] proposed a new HAR approach featuring separate spatial and temporal feature extraction phases. The model utilized preprocessing techniques, spatial and temporal blocks, and attention mechanisms, achieving high F1-scores across multiple datasets. Xia et al. [21] used a hybrid LSTM-CNN. Wang et al. [22] proposed an attention-based HAR method for weakly labeled data. The model leverages spatial-temporal feature integration and attention mechanisms to focus on relevant activity data, improving performance on noisy, weakly labeled datasets compared to CNN and LSTM-based approaches.

1D-CNNs stand out in their ability to effectively analyze time-series data, characterized by a single sequence of values. Unlike 2D-CNNs, which process data in two dimensions, 1D CNNs have fewer parameters, rendering them more efficient [23]. Compared to RNNs, commonly employed for time-series data analysis as well, 1D CNNs offer computational efficiency and simplicity in training. Moreover, they mitigate the vanishing gradient issue often encountered in LSTMs [24,25]. Lego filters [26,27], CondConv [28], and the matched filter CNN classifier [29] are lightweight deep learning approaches for that outperform conventional models. These methods utilize modular filter units, dynamic expert kernels, and signal processing techniques to enhance accuracy and computational efficiency on diverse datasets. CNNs can extract spatial and temporal features for human action recognition. Larger temporal filters capture long-term patterns, while smaller ones excel at short-term changes. Filter size selection is a critical hyperparameter balancing performance and computational complexity.

Graph Neural Networks (GCNs) known for modeling complex interactive activities are also reported for HAR [30,31,32,33,34,35]. Ghalan and Aggarwal [30] proposed a novel ensemble model, Graph Engineered EnsemCNN HAR (GE-EnsemCNN-HAR), combining CNNs with GCNs for improved classification of complex activities.Yang et al. [31] presented the Graph Domain Adaptation (GDA) network, a novel approach for sensor-based HAR that enhances model generalization, especially with limited data. By leveraging a graph neural network with adaptive learning and a local residual structure, the GDA network effectively captured non-Euclidean relationships in sensor signals. Ref. [32] introduced MG-WHAR, a novel method for wearable human activity recognition (WHAR) that models relationships among multi-sensors using graph structures. By constructing three types of graphs—based on body structure, sensor modality, and data patterns—MG-WHAR leverages multi-graph convolutional networks to enhance feature interactions and improve model performance. Belal et al. [33] explored HAR using sensory data and demonstrated the effectiveness of feature fusion for improving recognition accuracy. By employing a Parameter-Optimized Multi-Stage Graph Convolutional Network (PO-MS-GCN) and a Transformer. The study highlighted the limitations of existing models in capturing both spatial and temporal features. Duhme et al. [34] introduced Fusion-GCN, a method for multimodal action recognition that integrated various sensor data modalities into a graph for training with a GCN. By incorporating sensor measurements through additional node attributes or new nodes, Fusion-GCN flexibly fused RGB sequences, inertial measurements, and skeleton sequences. Huang et al. [35] introduced a deep framework for micro-gesture classification that utilizes ensemble models based on hypergraph-convolution transformers. The proposed approach enhanced the self-attention mechanism to better capture complex correlations within the skeleton data. Furthermore, the method employed data grouping and model ensemble techniques to address the challenges posed by imbalanced datasets.

Residual networks (ResNets) [36], a CNN variant with skip connections, have shown strong performance in using IMU data [20]. Their ease of training, ability to learn from smaller datasets, and adaptability to data changes are key strengths [37]. However, gradient noise can slow convergence and limit performance in certain tasks. Gated recurrent units (GRUs) [38] and LSTM [25], RNN variants, are strong for sequential data but face gradient issues. Residual connections in ResNets or transformers that leverage past/future context can mitigate these challenges in HAR. BiLSTM [39] networks are variants of LSTM, offering clear advantages. By processing the data in both forward and backward directions, they can capture both past and future context, which is essential for understanding and predicting actions. However, implementing BiLSTMs for HAR also requires more computational resources due to the need to process data in both directions [40]. Hybrids of CNNs with both GRUs and LSTMs have been also utilized for HAR [21,41,42]. These hybrid approaches leverage the strengths of CNNs in extracting spatial features and RNN’s sequential learning capabilities; to identify spatial and temporal patterns in the data effectively. LSTMs are exceptionally skilled at modeling long-term dependencies in sequential data, while GRUs are simpler versions of LSTMs that offer similar capabilities with fewer parameters [43], and less memory [42].

Zhang et al. [44] and Hassan et al. [9] both explored the use of deep belief networks (DBN), for HAR. While Zhang et al. [44] recommended DBNs for real-time activity recognition, Hassan et al. [9] found that DBNs outperformed shallow classification methods like ANN and SVM, achieving the highest recognition rate and accuracy.

Human actions usually involve long-term space-time interactions [45]. The use of transformers in human action recognition is due to attention mechanisms that better suppress redundancy and better model long-range interactions. Luptáková et al. [46] explored using transformer models for recognizing human activities through time-series data from wearable sensors. Employing the self-attention mechanism, the transformer model processed sequences effectively without recurrent structures. The study also employed data augmentation techniques to artificially expand the training dataset, thereby enhancing the model’s generalization capabilities. When comparing results on leaderboards, CNN models are still preferred. However, vision transformer-based models outperform CNN recognition in terms of accuracy, which is a crucial factor for human action recognition. LIMU-BERT [42], inspired by the bidirectional encoder representations from transformers (BERT) [47], can extract features from IMU data, but faces issues with transfer learning and handling rare instances, limiting its utility as a pre-trained model for HAR. The MobileHART model [48], a combination of transformers and CNNs, aims to be lightweight for smartphone deployment. However, it has a larger parameter count compared to other lightweight HAR models explored in the literature.

Yet, the computationally intensive nature of deep learning models hinders their implementation on resource-constrained platforms. Recurrent Neural Networks like LSTMs excel at modeling temporal dependencies but struggle with spatial data, while Convolutional Neural Networks excel at spatial feature extraction but lack temporal awareness [21,41,42,49]. Bidirectional LSTMs address this by capturing long-term dependencies by processing inputs in both forward and backward directions [40]. We investigate whether applying data augmentation techniques like time reversal [50] can emulate the contextual learning of BiLSTMs, potentially leading to more efficient HAR models. Table A1 provides a concise summary of the performance of various models from reviewed scholarly works along each dataset.

The 1DCNN-ResBiLSTM-Attention model [14] is a deep learning architecture that combines three components: a 1D CNN, residual BiLSTM, and attention mechanisms. Its primary goal is to improve the accuracy of similar action classification tasks. However, the integration of the additive operation with the BiLSTM increases the number of parameters in the model, which raises the question of whether it is possible to replace the BiLSTM with other components to reduce the number of parameters and keep the model lightweight while maintaining its performance. Can we approximate the nature of BiLSTM with the data flipping augmentation technique to read the input from two directions? Specifically, the following are the anticipated contributions of the manuscript:

- The paper investigates replacing the computationally intensive BiLSTM component in a HAR model with a combination of standard and residual LSTMs, as well as convolutional networks, to reduce the number of parameters and maintain model performance on resource-constrained devices.
- The study explores using data flipping augmentation to replicate the bidirectional context awareness provided by BiLSTMs, aiming to achieve similar performance with lower computational demands.
- The proposed modifications are evaluated on multiple datasets (i.e., UCI-HAR, WISDM, and KU-HAR) collected from mobile phone sensors (i.e., gyroscope, accelerometer) to demonstrate the effectiveness of the proposed methods.

## 2. Materials and Methods

The hybrid 1DCNN-ResBiLSTM-Attention model has demonstrated excellent HAR performance. In this work, we investigate: (1) whether ResBiLSTM can be replaced by lighter components, (2) methods to maintain model efficiency while preserving performance, and (3) whether BiLSTM’s contextual nature can be approximated through data augmentation. Section 2.1 provides an overview of the model, followed by the evaluation procedure in Section 2.2. A detailed description of the datasets used in the investigation is next presented in Section 2.3. Finally, the hyperparameter tuning protocol is then described in Section 2.4.

We utilized Python due to its extensive collection of machine-learning supportive libraries. Additionally, we employed the TensorFlow [51] and Keras [52] Python libraries to implement the model architectures. To accelerate the training process, we leveraged Google Colaboratory Pro [53], which provided access to powerful GPU resources such as K80, P100, T4, and 32 GB of RAM. Furthermore, we employed grid search (GridSearchCV)  [54] techniques for hyperparameter tuning. The code is available publicly at https://github.com/deema-mohammed10/G3_P8_DL-based-framework-for-HAR_Code (accessed on 21 August 2024).

### 2.1. Overview of the Proposed Models and Experimental Procedure

**Figure 1:**
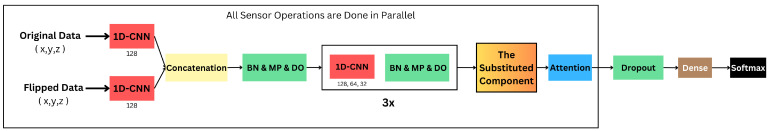
Proposed model architecture for single sensor and data augmentation.

In refining our HAR approach, we integrated time reversal data augmentation to enhance input diversity. The method (Figure 1) processes original and flipped data through parallel 1D-CNN pathways, followed by concatenation, batch normalization (BN), max pooling (MP), and dropout (DO) to mitigate overfitting. Let $X=[x_{1},x_{2},\ldots ,x_{n}]$ be the original time series data of length *n*. Let *w* be the window size. Let *t* be the current time index, where 1≤t≤n.

The window data flipping operation can be expressed as:

$$X_{\mathrm{flipped}}\left[t\right]=x_{\mathrm{end}},x_{\mathrm{end}-1},\ldots ,x_{\mathrm{start}+1}$$

where:

$$\mathrm{start}=\max(1,\left\lfloor t-\frac{w}{2}\right\rfloor )$$

$$\mathrm{end}=\min(n,\left\lfloor t+\frac{w}{2}\right\rfloor )$$

This formula determines the start and end indices of the window of size *w* centered at *t*. It then slices the values from the end to the start of the window and assigns them to $X_{\mathrm{flipped}}\left[t\right]$, effectively flipping the window data.

The original and flipped data are processed through parallel 1D-CNN pathways. Let $h_{\mathrm{cnn}1}\left(x\right)$ and $h_{\mathrm{cnn}1}\left(x_{\mathrm{flip}}\right)$ represent the outputs of the 1D-CNN for the original and flipped data, respectively. The outputs from the parallel 1D-CNN pathways are concatenated:

$$h_{concat}=\mathrm{Concat}\left(h_{\mathrm{cnn}1}\left(x\right),h_{\mathrm{cnn}1}\left(x_{\mathrm{flip}}\right)\right)$$

The concatenated output is then batch normalized, followed by max pooling and dropout:

$$h_{\mathrm{bn}}=\mathrm{BatchNorm}\left(h_{concat}\right)$$

$$h_{\mathrm{mp}}=\mathrm{MaxPool}\left(h_{bn}\right)$$

$$h_{\mathrm{do}}=\mathrm{Dropout}\left(h_{mp}\right)$$

**Figure 2:**
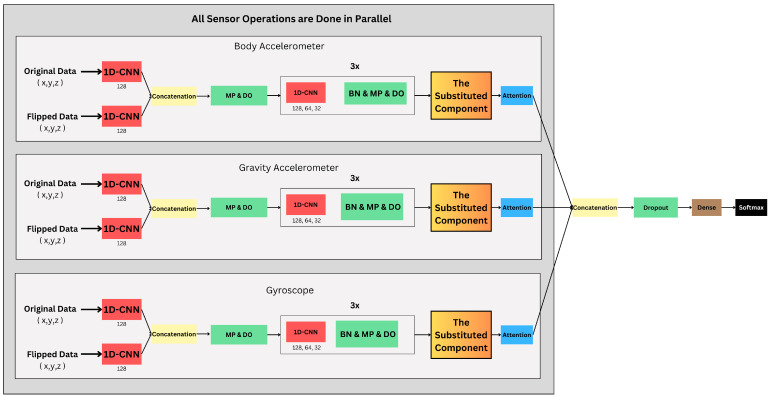
Proposed model architecture for multi-sensor and data augmentation.

We then tested substituting different components for the central layer. An attention mechanism prioritizes significant features for nuanced analysis. This multi-branch model (Figure 2) accommodates datasets with varying sensor types, such as UCI-HAR, WISDM, and KU [11,14]. The number of branches (each processing data from a different sensor) depends on the dataset used.

The proposed model applies the time reversal data augmentation, which vertically flips the input data of a single window using the flip function from the NumPy library. After passing through the 1D-CNN, the inputs will be concatenated and fed into another sequence of layers of 1D-CNNs, MP, and DO. We then test various “Substituted Components”—1D-CNN, LSTM, BiLSTM, and their residual variants—to determine their efficacy. The 1D-CNN was initially chosen as it had demonstrated promising results for signal data processing [23]. However, CNNs inherently lack the ability to naturally handle temporal information, requiring the use of overlapping windows. In contrast, LSTMs are well-suited for temporal data and are also computationally lighter. Additionally, we evaluated BiLSTM and ResBiLSTM [14], which were part of the original base model, as well as residual versions of both CNN and LSTM, to provide a fair comparison against the ResBiLSTM architecture.

Residual versions use a point-wise additive operation between sequential layers, rather than complex residual connections, to maintain model efficiency. For example, the output from a residual LSTM (ResLSTM) can be expressed as:

$$h_{\mathrm{resLSTM}}=\mathrm{LSTM}\left(h_{\mathrm{do}}\right)+h_{\mathrm{do}}$$

Instead of a more complex residual connection that would inevitably increase the number of parameters, these “Res” models use an explicit add function to combine the outputs of two sequential layers. The output is passed through an attention mechanism to improve activity recognition accuracy. An attention mechanism is used to prioritize significant features. Let $\alpha_{i}$ be the attention weight for the *i*-th feature, and $h_{\mathrm{final}}$ be the weighted sum of features:

$$\alpha_{i}=\frac{\exp(e_{i})}{\sum_{j}\exp\left(e_{j}\right)}$$

$$h_{\mathrm{final}}=\sum_{i}\alpha_{i}h_{i}$$

where $e_{i}$ is the attention score for the *i*-th feature, often computed as:

$$e_{i}=\tanh\left(W_{h}h_{i}+b_{h}\right)$$

with $W_{h}$ and $b_{h}$ being learnable parameters.

Finally, the multi-sensor branch outputs are concatenated and processed through dropout, dense, and softmax layers. For multi-sensor data, let $h_{\mathrm{branch}1},h_{\mathrm{branch}2},\ldots ,h_{\mathrm{branchn}}$ be the outputs of different branches processing data from different sensors. These outputs are concatenated:

$$h_{\mathrm{multi}}=\mathrm{Concat}\left(h_{\mathrm{branch}1},h_{\mathrm{branch}2},\ldots ,h_{\mathrm{branchn}}\right)$$

The concatenated output from the multi-sensor branches is processed through dropout, dense, and softmax layers for classification:

$$h_{\mathrm{do}\_\mathrm{final}}=\mathrm{Dropout}\left(h_{\mathrm{multi}}\right)$$

$$h_{\mathrm{dense}}=\mathrm{Dense}\left(h_{\mathrm{do}\_\mathrm{final}}\right)$$

$$y_{\mathrm{pred}}=\mathrm{Softmax}\left(h_{\mathrm{dense}}\right)$$

### 2.2. Evaluation Procedure

In total, we have six models to test out divided into three groups as shown in Table 1, with the hyperparameters that were tuned. Group B and C can be represented in Figure 1 and Figure 2; the choice is based on the number of sensors in the used dataset. The components of both groups are the same apart from “The Substituted Component”, which is substituted with ResLSTM, LSTM, ResCNN, or CNN based on the desired group. On the other hand, models in Group A only have the original data as input.

Given that it is a multi-class classification problem, the performance metrics are calculated for each class independently and then macro-averaged (i.e., gives equal weight to each class) across all classes. Let $TP_{c}$ and $TN_{c}$ denote the numbers of true positives and true negatives, respectively, for class *c*, and $FP_{c}$ and $FN_{c}$ denote the numbers of false positives and false negatives, respectively, for class *c*. Additionally, let *C* represent the total number of classes. Accuracy (Acc) measures the proportion of correctly classified instances across all classes (see Equation (1)). Precision (*P*) assesses the model’s ability to correctly predict positive instances for each class (see Equation (2)). Recall (*R*) or Sensitivity evaluates the model’s ability to identify all positive instances for each class (see Equation (3)). The F1-score combines precision and recall into a single metric, providing a harmonic mean of the two (see Equation (4)). We also assess the model’s complexity by counting the parameters it utilizes, and the model training time in seconds.

(1) $$Acc=\frac{1}{C}\sum_{c=1}^{C}\frac{TP_{c}+TN_{c}}{TP_{c}+TN_{c}+FP_{c}+FN_{c}}$$

(2) $$P=\frac{1}{C}\sum_{c=1}^{C}\frac{TP_{c}}{TP_{c}+FP_{c}}$$

(3) $$R=\frac{1}{C}\sum_{c=1}^{C}\frac{TP_{c}}{TP_{c}+FN_{c}}$$

(4) $$F1=\frac{1}{C}\sum_{c=1}^{C}2\times \frac{P_{c}\times R_{c}}{P_{c}+R_{c}}$$

During the execution phase, we will employ a grid search for hyperparameter tuning with a five-fold cross-validation to find the best parameters for the six models. The evaluation will be based on the loss function for hyperparameter tuning. Furthermore, we will diligently evaluate its performance utilizing methodologies, including the confusion matrix, to ascertain the recall and F1-score.

Finally, we will evaluate the number of parameters in our model and compare them to those of the 1D CNN-ResBiLSTM-Attention model [14] and other related work [19,21,41].

### 2.3. Datasets

In this research, three HAR datasets (i.e., University of California, Irvine HAR dataset (UCI-HAR) [55], wireless sensor data mining dataset (WISDM) [56], Khulna University dataset (KU-HAR) [57]) are used for the evaluation of the proposed methods (see Table 2 for detailed summaries). The selection of these datasets is based on several criteria to ensure a comprehensive evaluation of the proposed models. First, the chosen datasets encompass a variety of sensors, including accelerometers and gyroscopes, which are commonly found in smartphones, allowing the assessment of model performance across different sensor inputs. Second, each dataset includes a wide range of activity types (i.e., actions of both static and periodic nature), from basic movements like walking and sitting to more complex actions like jumping and playing table tennis, ensuring the models are tested on both simple and complex activities. Additionally, the UCI-HAR dataset offers a balanced class distribution, while WISDM and KU-HAR present imbalanced distributions, providing a robust evaluation under different data distribution scenarios. Third, these datasets are widely recognized and frequently used in HAR research, allowing for easier comparison with existing state-of-the-art methods. Finally, the datasets vary significantly in the number of instances and subjects, ranging from 10,299 instances and 30 subjects in UCI-HAR to 1,098,207 instances and 36 subjects in WISDM, and 20,750 instances and 90 subjects in KU-HAR, ensuring the models are evaluated on both small and large-scale datasets, demonstrating their scalability and robustness.

Table 2 specifies the number of subjects (No. Sub), number of instances (No. Ins), number of classes, and the sensors employed, such as accelerometer (Accel.) and gyroscope (gyro.) for each dataset. Notably, these datasets were constructed from smartphone-mounted sensors. Furthermore, it lists the types of activities performed in each dataset. The UCI-HAR dataset has a good balance of classes, whereas the class distribution in the WISDM and KU-HAR datasets is not balanced.

### 2.4. Hyperparameter Tuning

We opted to tune all six models using the UCI-HAR dataset, as it provided several sensors which were the body accelerometer, gravity accelerometer, and gyroscope, in addition to its balanced nature. Exploring the different models mentioned before, which are “no data augmentation with the original ResBiLSTM model”, “no data augmentation with the BiLSTM model”, “data augmentation with the ResLSTM model”, “data augmentation with the LSTM model”, “data augmentation with the ResCNN model”, and “data augmentation with the CNN model”. Our focus was on tuning various aspects including activation function, dropout combination, learning rate, kernel size, and batch size as shown in Table 3. The $\left(a_{1},a_{2}\right)$ dropout pair denotes the dropout rates used for the first and second dropout blocks in Figure 1. Each model went through a 5-fold cross-validation for tuning, and evaluating metrics such as best accuracy, precision, recall, F1-score, and the number of parameters.

The outcome of tuning for the models and their respective best parameters are shown in Table 4. The best kernel sizes for the Data augmentation with ResCNN model and CNN model were 3 and 5, respectively. The selection of the best parameters was based on achieving a balance between the best accuracy and the number of parameters. This choice was informed by the well-balanced nature of the UCI-HAR dataset, where average accuracy is crucial, while lower parameters contribute to efficiency.

## 3. Results

Table 5, Table 6 and Table 7 present the results obtained by the different configurations on the UCI-HAR, WISDM, and KU-HAR datasets, respectively. Each table includes detailed metrics for various model configurations, highlighting the accuracy (Acc), precision (*P*), recall (*R*), F1-score (F1), the number of model parameters (Param), and the training time in seconds. The substituted component is denoted by (Sub. Comp.).

In our analysis of Table 5, Table 6 and Table 7, we observed that Group A, which includes BiLSTM and ResBiLSTM architectures, not only achieves the highest performance metrics but also demonstrates a substantial increase in the number of parameters compared to other models. This suggests that the higher performance of Group A comes at the cost of increased model complexity and computational requirements. Specifically, the ResBiLSTM often slightly outperforms the BiLSTM, which could be attributed to the additional parameters that might help us in learning the more nuanced features of the data.

Group B, comprising LSTM and ResLSTM, strikes a balance between performance and the number of parameters. These models have significantly fewer parameters than those in Group A, potentially making them more efficient in terms of computational resources while still maintaining good performance. The ResLSTM, which slightly outperforms the standard LSTM in most cases, shows how minor adjustments and additional parameters in the LSTM architecture can enhance performance without dramatically increasing complexity.

Group C, which includes CNN and ResCNN, consistently shows the lowest performance. However, it also tends to have fewer parameters, especially in the ResCNN variants. This highlights a crucial aspect: CNNs are less capable of effectively capturing temporal dependencies.

### 3.1. Performance Analysis for Results Obtained on the UCI-HAR Dataset

**Figure 3:**
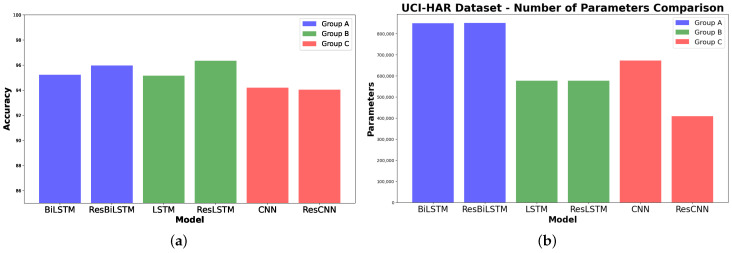
Performance comparison of the different models in each group on the UCI-HAR dataset. (**a**) Accuracy scores. (**b**) Number of parameters.

As shown in Figure 3a, the accuracy of Group A, which used BiLSTM base models without data augmentation, was compared to the results of Group B and Group C. Group B and Group C both utilized data augmentation techniques, with Group B coupling data augmentation with LSTM-based models and Group C pairing it with CNN models. The results for the ResLSTM model were similar to, or even higher than, the performance of the other groups. The ResLSTM model achieves the highest accuracy score among the evaluated models with an accuracy of 96.34%, as shown in Table 5. Additionally, the ResLSTM model has parameters that are lower than those of the CNN model, as shown in Figure 3b.

Compared with other models in the literature review, the 1DCNN-ResBiLSTM [14] model reported an accuracy obtained training and validation of 98.37%, but its test accuracy decreased to 95.96% (Group A). CNN-LSTM models [21,41] reported 97.89% and 95.8% test accuracies, respectively, higher than our LSTM and ResLSTM models in Group B. The confusion matrices and learning curves for each model provide detailed classification results and training dynamics, with early stopping used to determine the optimal number of epochs.

#### 3.1.1. Numerical Analysis of Group A Models

**Figure 4:**
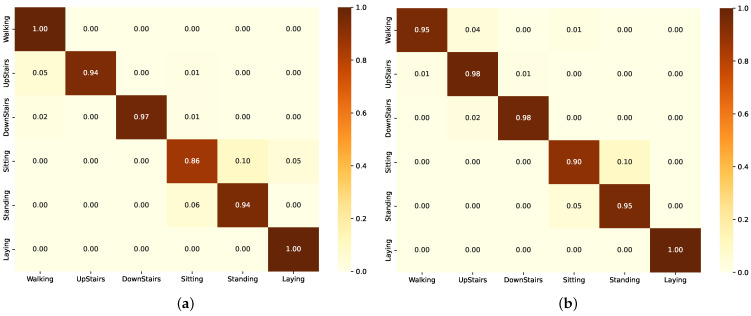
Confusion matrix of the model in Group A as evaluated on the UCI-HAR dataset. (**a**) Using BiLSTM component. (**b**) Using ResBiLSTM component.

The confusion matrix for the BiLSTM model in Figure 4a shows high accuracy in identifying Walking and Laying with no misclassifications. However, Upstairs and Downstairs have some misclassifications, particularly with Walking. ’Sitting’ is often confused with Standing and Laying, while Standing has instances misclassified as Sitting. The confusion matrix for the ResBiLSTM model (Figure 4b) shows high accuracy in identifying Laying with no misclassifications. However, Walking, Upstairs and Downstairs have some misclassifications, particularly with Walking. Sitting and Standing are often confused with each other.

**Figure A1:**
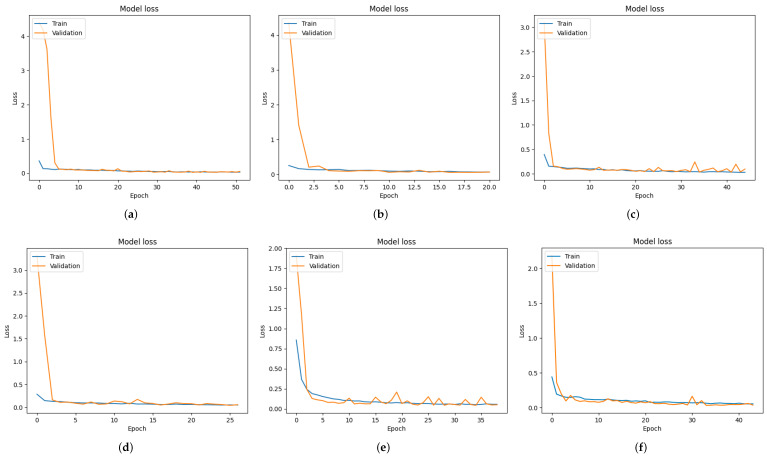
Training loss curves for the implemented models using UCI-HAR dataset. (**a**) BiLSTM, (**b**) ResBiLSTM, (**c**) LSTM, (**d**) ResLSTM, (**e**) CNN, (**f**) ResCNN.

**Figure A2:**
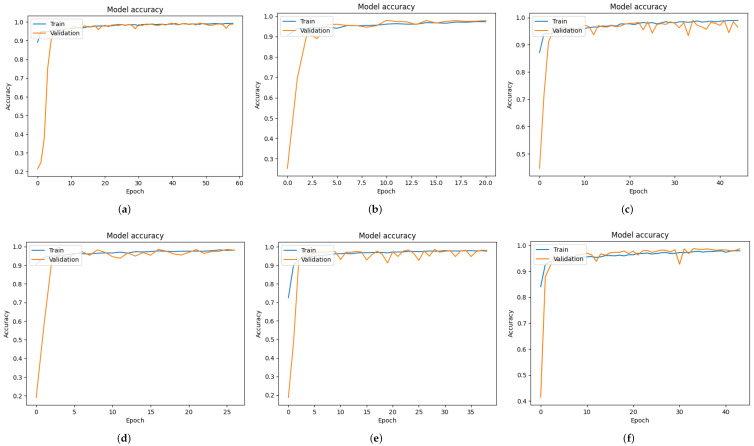
Training accuracy curves for the implemented models using UCI-HAR dataset. (**a**) BiLSTM, (**b**) ResBiLSTM, (**c**) LSTM, (**d**) ResLSTM, (**e**) CNN, (**f**) ResCNN.

The learning curve of the BiLSTM model in Group A using the UCI-HAR dataset in Figure A1 and Figure A2 show both model loss and accuracy over 60 epochs. The training and validation loss curves converge rapidly, stabilizing near zero, reflecting efficient learning and minimal error. Similarly, the accuracy curves for both training and validation data plateau near 1.0, indicating high accuracy and good generalization to unseen data.

The learning curve of the ResBiLSTM model using the UCI-HAR dataset in Figure A1 and Figure A2 shows both model loss and accuracy over 30 epochs. The training and validation loss curves converge rapidly, similar to the rest of the models.

#### 3.1.2. Numerical Analysis of Group B Models

**Figure 5:**
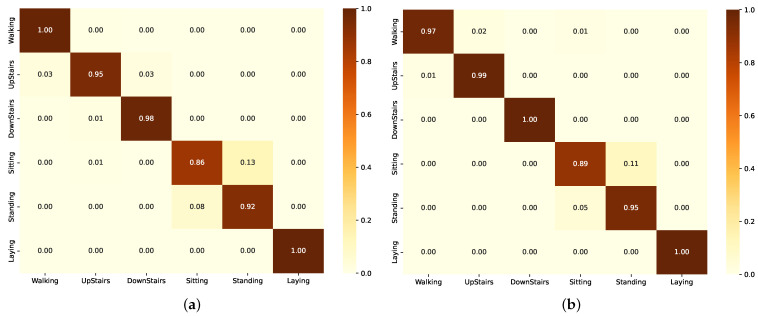
Confusion matrix of the model in Group B as evaluated on the UCI-HAR dataset. (**a**) Using LSTM component. (**b**) Using ResLSTM component.

The confusion matrix for the LSTM model in Figure 5a shows high accuracy in identifying Laying with no misclassifications. However, sitting and standing are often confused. The confusion matrix for the ResLSTM model in Figure 5b shows high accuracy in identifying Laying and Downstairs with no misclassifications. Sitting and Standing are often confused with each other.

In Figure A1 and Figure A2 the learning curve of the LSTM model using the UCI-HAR dataset shows both model loss and accuracy over 25 epochs. The training and validation loss curves converge rapidly similar to the last models.

In Figure A1 and Figure A2 the learning curve of the ResLSTM model using the UCI-HAR dataset shows both model loss and accuracy over 60 epochs. The training and validation loss curves converge rapidly like the last models.

#### 3.1.3. Numerical Analysis of Group C Models

**Figure 6:**
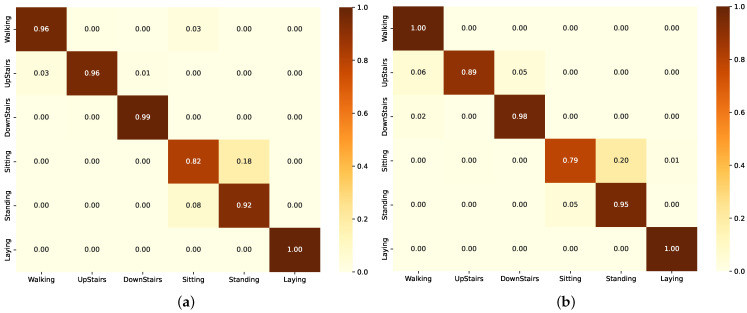
Confusion matrix of the model in Group C as evaluated on the UCI-HAR dataset. (**a**) Using CNN component. (**b**) Using ResCNN component.

The confusion matrix for the CNN model in Figure 6a shows high accuracy in identifying Laying with no misclassifications. Sitting and Standing are often confused with each other. The confusion matrix for the ResCNN model in Figure 6b shows high accuracy in identifying Laying and Walking with no misclassifications. Sitting and Standing are often confused with each other.

In Figure A1 and Figure A2 the learning curve of the CNN model using the UCI-HAR dataset shows both model loss and accuracy over 40 epochs. The training and validation loss curves converge rapidly like the last models.

In Figure A1 and Figure A2 the learning curve of the ResCNN model using the UCI-HAR dataset shows both model loss and accuracy over 40 epochs. The training and validation loss curves converge rapidly like the last models.

### 3.2. Performance Analysis for Results Obtained on the WISDM Dataset

The WISDM dataset is imbalanced since the “Walking” takes almost 39% of the class distribution, as opposed to 4% for the “Standing” class, for example [56]. The F1-score provides a crucial metric in this case.

**Figure 7:**
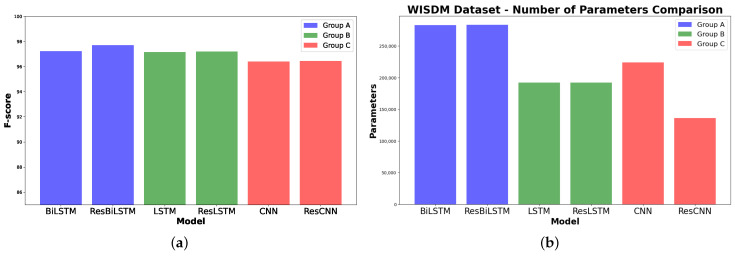
Performance comparison of the different models in each group using the WISDM dataset. (**a**) F1-scores. (**b**) Number of parameters.

The graphs in Figure 7 represent the F1-scores, and through it, we can see that (Group A) models demonstrated robust performance with the ResBiLSTM achieving the highest F1-score of 97.71% as also shown in Table 6. This illustrates the effectiveness of bidirectional architectures in grasping the complex temporal dependencies within the activity data. The standard BiLSTM also performed commendably with an F1-score of 97.23% (Table 6), reinforcing the capability of LSTM-based architectures in context recognition.

In contrast, (Group B) leveraged data augmentation, with the ResLSTM model as in Table 6 illustrating an F1-score of 97.20%, marginally lower than (Group A)’s top performer but significantly effective, demonstrating that residual connections can enhance LSTM’s performance by deepening the feature extraction process without excessive parameter increase.

Table 6 shows that (Group C) explored simpler CNN and ResCNN models, achieving F1-scores of 96.40% and 96.45% respectively. These results are competitive, especially when considering the computational efficiency of CNNs. The ResCNN’s slight edge over the standard CNN highlights the benefit of integrating residual learning to bolster feature learning capabilities.

Comparing these outcomes to other models reported in the literature, such as the GRU, INC, ResNets, CBAM, and attention mechanisms [20] with an F1-score of 99.12%. This ensemble approach using GRUs and attention mechanisms and combining the RNN, CNN, and Attention base components, as in our models in (Group B), showcases a 2% decrease in the F1-score.

#### 3.2.1. Numerical Analysis of Group A Models

**Figure 8:**
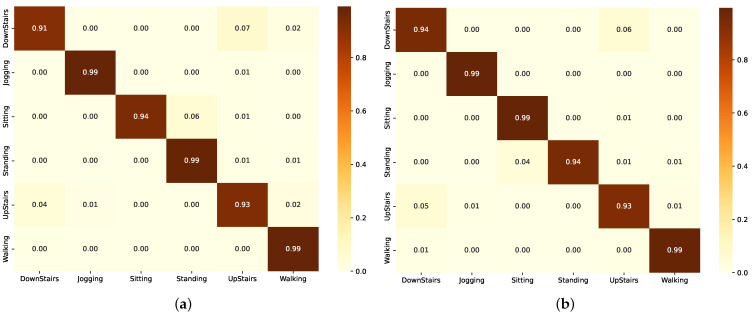
Confusion matrix of the model in Group A, as evaluated using the WISDM dataset. (**a**) Using BiLSTM component. (**b**) Using ResBiLSTM component.

The confusion matrix in Figure 8a shows the performance of the BiLSTM model across different activities. The model demonstrates high accuracy in identifying JOGGING and WALKING, with 1087 and 1271 correct classifications, respectively. However, there are some notable misclassifications, such as UPSTAIRS being confused with DOWNSTAIRS and WALKING, and SITTING being occasionally misclassified as STANDING. In Figure 8b, the confusion matrix of the ResBiLSTM model using the WISDM dataset shows several misclassifications, especially for UPSTAIRS, which is often confused with DOWNSTAIRS and WALKING. Despite high accuracy in identifying JOGGING and WALKING, there are notable errors, such as SITTING being misclassified as STANDING and vice versa. The unbalanced nature of the dataset highlights these misclassifications.

**Figure A3:**
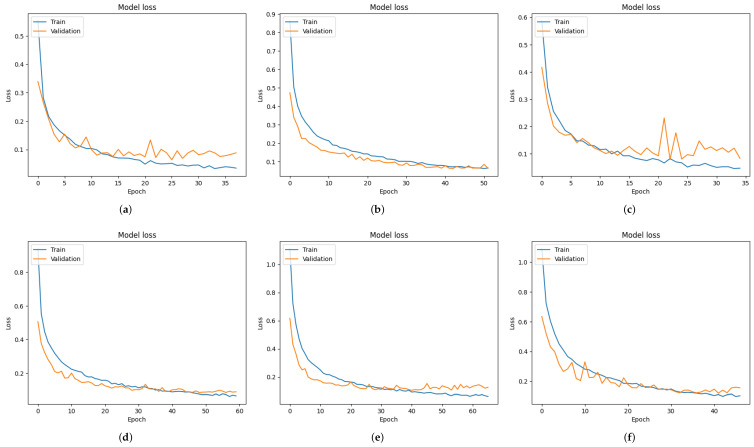
Training loss curves for the implemented models using WISDM dataset. (**a**) BiLSTM, (**b**) ResBiLSTM, (**c**) LSTM, (**d**) ResLSTM, (**e**) CNN, (**f**) ResCNN.

**Figure A4:**
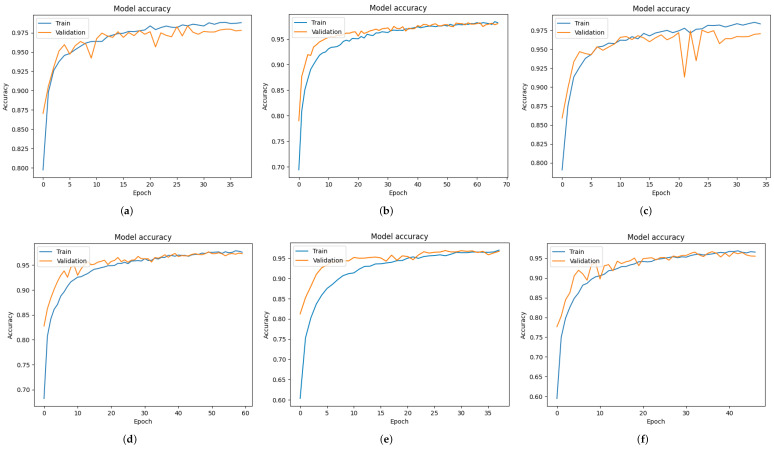
Training accuracy curves for the implemented models using WISDM dataset. (**a**) BiLSTM, (**b**) ResBiLSTM, (**c**) LSTM, (**d**) ResLSTM, (**e**) CNN, (**f**) ResCNN.

The learning curves of the BiLSTM model using the WISDM dataset in Figure A3 and Figure A4 show both model loss and accuracy over 20 epochs. The training and validation loss curves converge rapidly, stabilizing near 0.1, reflecting efficient learning and minimal error. Similarly, the accuracy curves for both training and validation data plateau near 0.975, indicating high accuracy and good generalization to unseen data.

The learning curve in Figure A3 and Figure A4 of the ResBiLSTM model using the WISDM dataset shows both model loss and accuracy over 70 epochs. The training and validation loss curves converge rapidly, stabilizing near 0.1, reflecting efficient learning and minimal error. Similarly, the accuracy curves for both training and validation data plateau near 0.98, indicating high accuracy and good generalization to unseen data.

#### 3.2.2. Numerical Analysis of Group B Models

**Figure 9:**
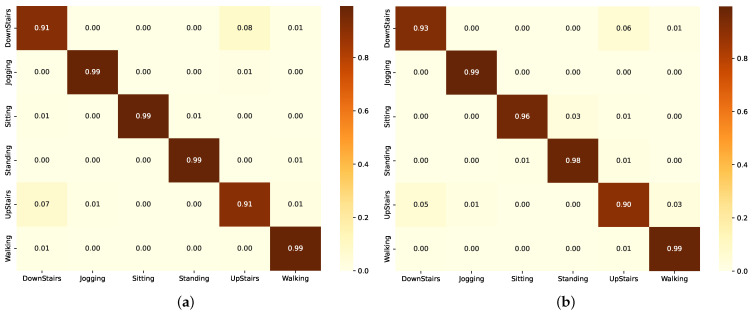
Confusion matrix of the model in Group B as evaluated on the WISDM dataset. (**a**) Using LSTM component. (**b**) Using ResLSTM component.

The confusion matrices of the LSTM and ResLSTM models on the WISDM dataset (Figure 9) reveal misclassifications, particularly for UPSTAIRS, which is often confused with DOWNSTAIRS and WALKING. Despite high accuracy for JOGGING and WALKING, there are errors like SITTING being misclassified as STANDING, likely due to dataset imbalance.

The LSTM model’s learning curves on the WISDM dataset (Figure A3 and Figure A4) show rapid convergence of training and validation loss near 0.1, with both training and validation accuracy plateauing at around 0.97, indicating effective learning and good generalization to unseen data. A similar observation can be drawn from Figure A3 and Figure A4 for the ResLSTM model.

#### 3.2.3. Numerical Analysis of Group C Models

**Figure 10:**
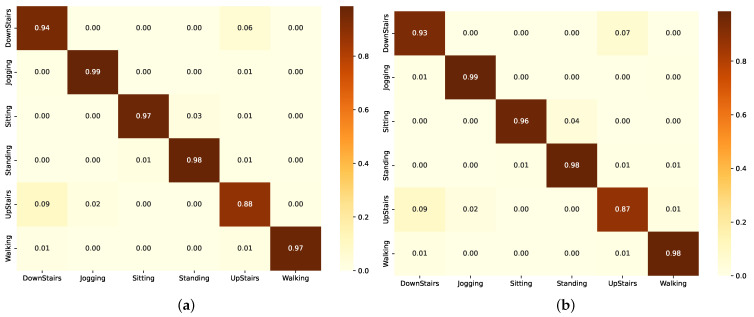
Confusion matrix of the model in Group C as evaluated on the WISDM dataset. (**a**) Using CNN component. (**b**) Using ResCNN component.

The CNN and ResCNN models’ confusion matrices on the WISDM dataset (Figure 10) show difficulties classifying UPSTAIRS, often confused with DOWNSTAIRS and WALKING. While accurate for JOGGING and WALKING, there are significant errors, such as SITTING being misclassified as STANDING, likely due to dataset imbalance.

The CNN and ResCNN models’ learning curves on the WISDM dataset in Figure A3 and Figure A4 show rapid convergence of training and validation loss near 0.1, with accuracy plateauing around 0.97, indicating effective learning and good generalization, despite some fluctuations in the ResCNN’s validation accuracy.

### 3.3. Performance Analysis for Results Obtained on the KU-HAR Dataset

The KU-HAR dataset, similar to the WISDM dataset, suffers from an imbalanced class distribution [57]. Therefore, the F1-score is utilized for evaluating model performance.

**Figure 11:**
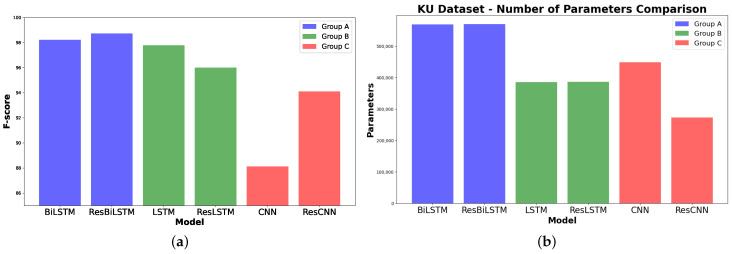
Performance comparison of the different models in each group on the KU-HAR dataset. (**a**) F1-scores. (**b**) Number of parameters.

Group A models including BiLSTM and ResBiLSTM deliver exceptional performance with F1-scores of 98.22% and 98.72%, respectively (Figure 11). Group B which includes LSTM and its enhanced variant, ResLSTM, shows notable effectiveness with F1-scores of 97.78% and 96.00%, respectively. Data augmentation was employed to potentially enhance model performance by augmenting the dataset with time-reversed sequences. Group C, comprising basic CNN and ResCNN models, demonstrates their commendable performance with F1-scores of 88.12% and 94.10%, respectively. The noticeable improvement in the ResCNN model underscores the benefits of integrating additive operations between layers to enhance feature extraction capabilities. Data augmentation techniques were similarly utilized in this group to improve model robustness and performance.

Our model achieves an F1-score of 98.72% on the KU-HAR dataset, slightly exceeding the previously reported 98.16% for an attention-based Residual BiLSTM model [14]. This improvement indicates effective learning of complex temporal patterns with high accuracy and reasonable computational load. In contrast, a Transformer-based model [46] attained a higher 99.2% F1-score, but requires significantly more resources, making our model more practical for applications prioritizing efficiency over absolute peak performance.

#### 3.3.1. Numerical Analysis of Group A Models

**Figure 12:**
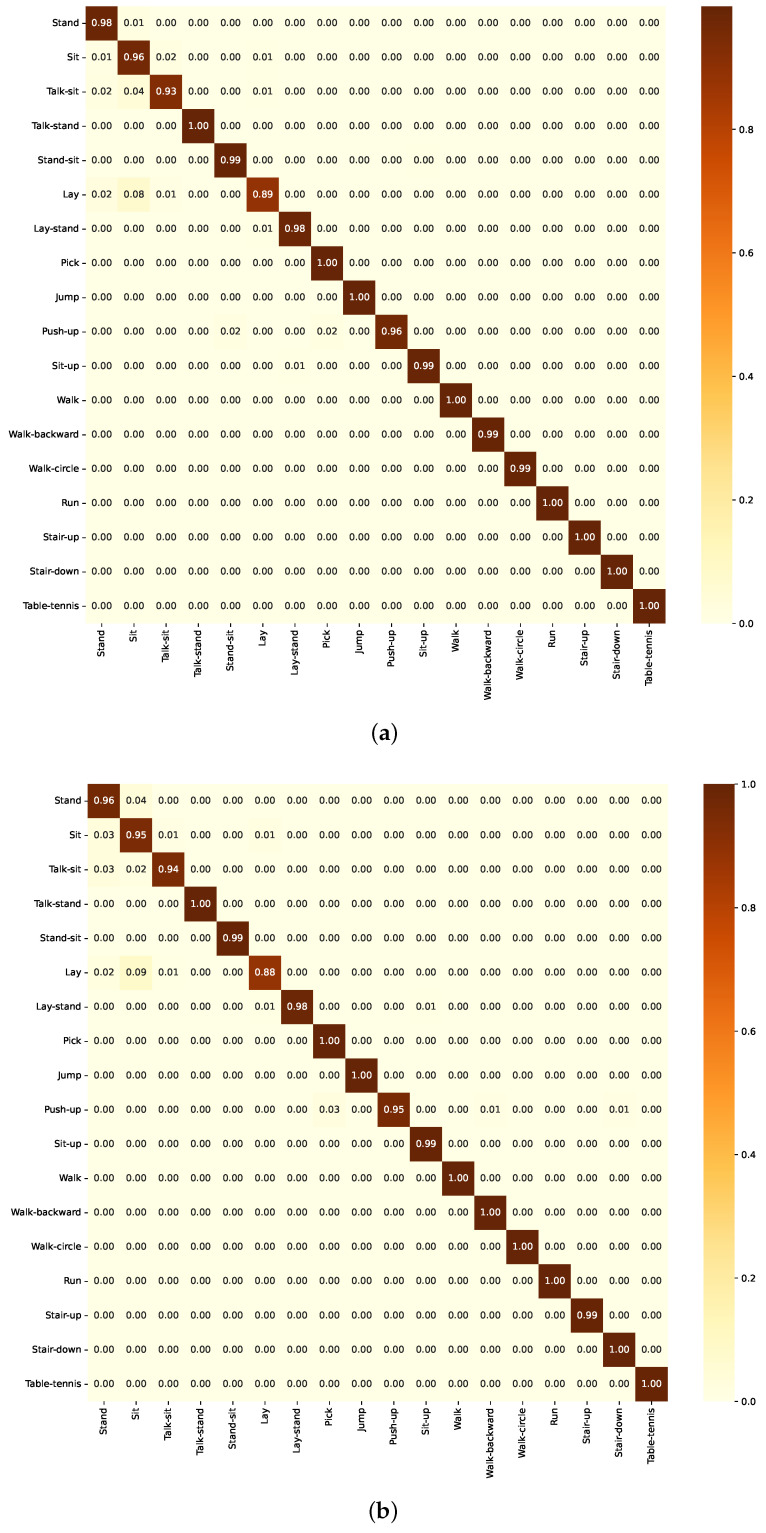
Confusion matrix of the model in Group A as evaluated on the KU-HAR dataset. (**a**) Using BiLSTM component. (**b**) Using ResBiLSTM component.

For the BiLSTM model, the confusion matrix (Figure 12a) indicates generally high performance, with high true positive rates. However, there are some misclassifications between similar activity classes. Similarly, the ResBiLSTM model also demonstrates reasonable performance, with the confusion matrix (Figure 12a) again exhibiting high true positive rates for most activity classes, but facing some challenges in distinguishing closely related activities.

**Figure A5:**
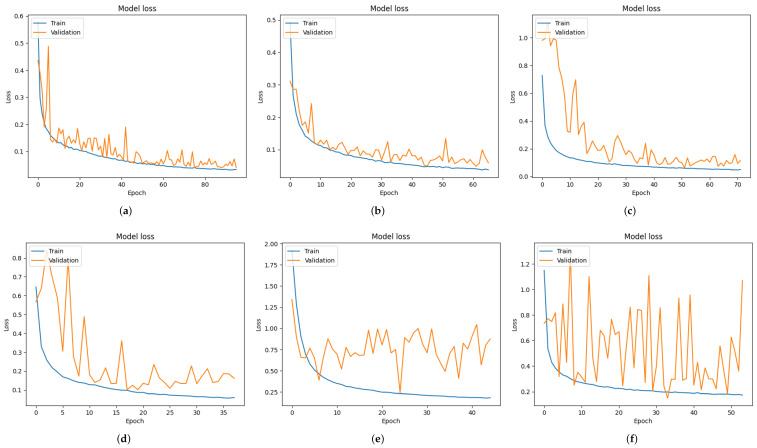
Training loss curves for the implemented models using KU-HAR dataset. (**a**) BiLSTM, (**b**) ResBiLSTM, (**c**) LSTM, (**d**) ResLSTM, (**e**) CNN, (**f**) ResCNN.

**Figure A6:**
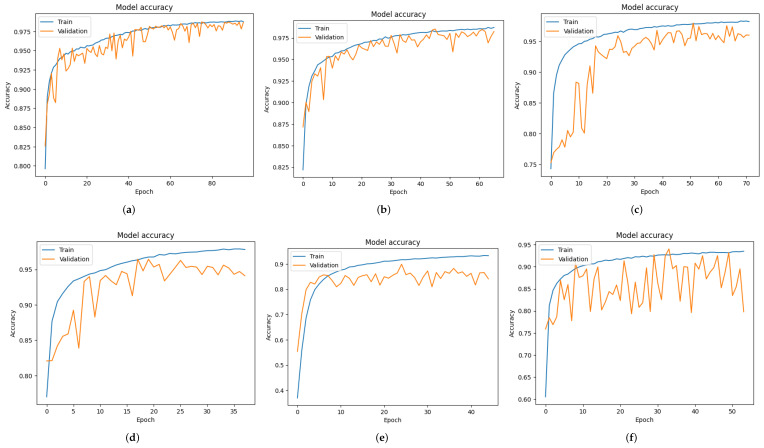
Training accuracy curves for the implemented models using KU-HAR dataset. (**a**) BiLSTM, (**b**) ResBiLSTM, (**c**) LSTM, (**d**) ResLSTM, (**e**) CNN, (**f**) ResCNN.

The model loss plot in Figure A5 exhibits significant fluctuations in the early epochs, with the training loss spiking dramatically before gradually stabilizing. The validation loss also shows substantial oscillations, particularly in the initial stages. Looking at the model accuracy plot in Figure A6, the training and validation accuracy curves start around 0.8 and steadily improve, eventually reaching their peak performance of around 0.98 after approximately 60 epochs.

The ResBiLSTM loss plot (Figure A5) exhibits significant fluctuations in the early epochs, with the training loss spiking dramatically before gradually stabilizing. The validation loss also shows substantial oscillations, particularly in the initial stages. Looking at the model accuracy plot (Figure A6), the training and validation accuracy curves start around 0.87 and gradually improve, eventually reaching their peak performance of around 0.99 after approximately 100 epochs.

#### 3.3.2. Numerical Analysis of Group B Models

**Figure 13:**
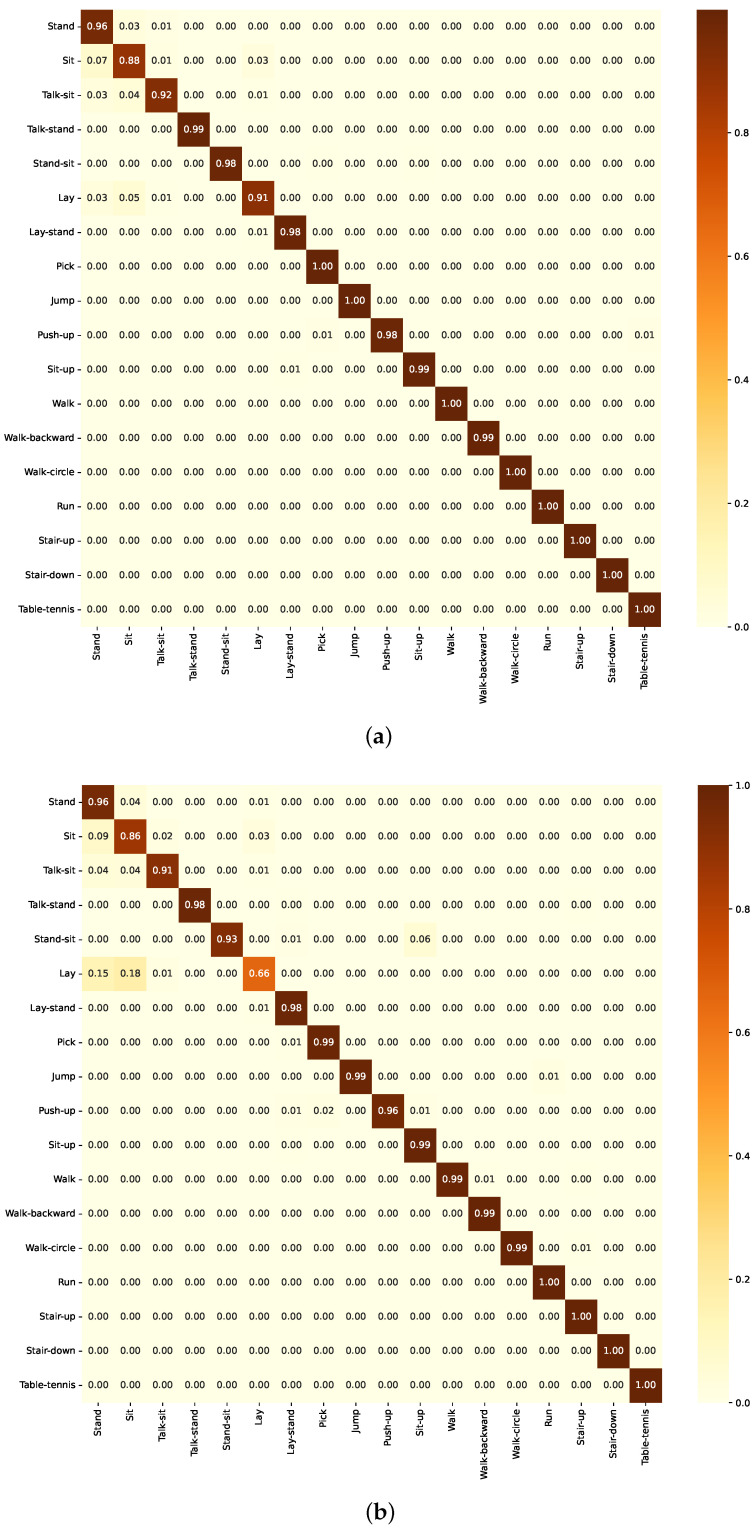
Confusion matrix of the model in Group B as evaluated on the KU-HAR dataset. (**a**) Using LSTM component. (**b**) Using ResLSTM component.

For the LSTM model, the confusion matrix (Figure 13a) generally shows strong true positive rates along the diagonal. However, there are some misclassifications between similar activity classes such as “Sit-up” and “Walk-backward”. For the ResLSTM model, the confusion matrix (Figure 13b) demonstrates the model’s strong classification abilities, with high true positive rates along the diagonal, indicating accurate recognition of most activity classes. However, there are some classifications between activities.

The learning curves for the LSTM and ResLSTM (Figure A5 and Figure A6) models exhibit significant early fluctuations, with training loss spiking before stabilizing, and validation loss showing substantial oscillations. However, the accuracy curves steadily improve, reaching peak performance around 0.98 and 0.97 after 80 and 40 epochs respectively. This pattern indicates the models effectively learned complex patterns, despite the initial instability in loss.

#### 3.3.3. Numerical Analysis of Group C Models

**Figure 14:**
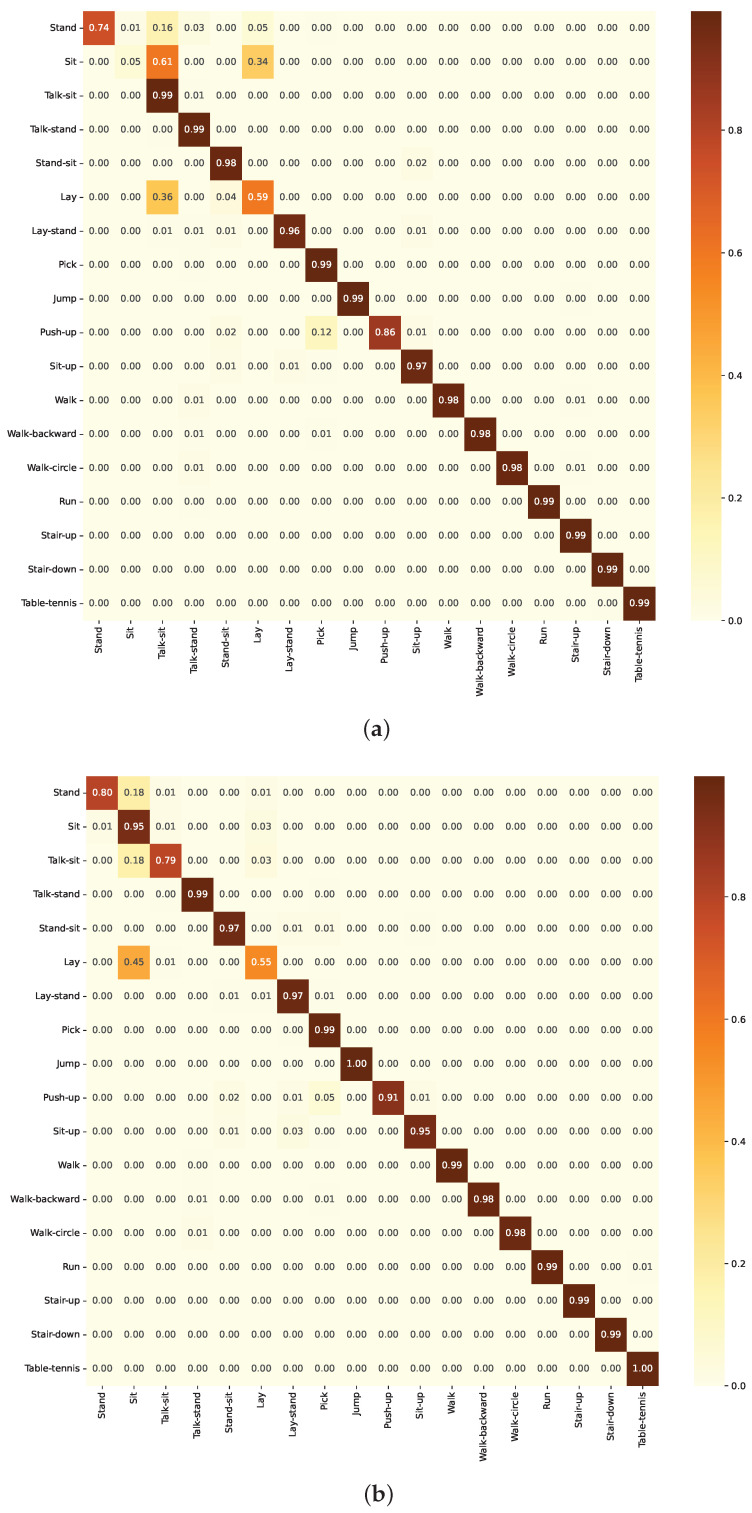
Confusion matrix of the model in Group C as evaluated on the KU-HAR dataset. (**a**) Using CNN component. (**b**) Using ResCNN component.

The confusion matrix for the CNN model (Figure 14a) and the ResCNN model (Figure 14a) demonstrates strong performance on the KU-HAR dataset. The matrix shows high true positive rates along the diagonal, indicating the model’s ability to accurately classify most activity classes. However, there are some noticeable misclassifications, especially between similar activities such as “Sit-up” and “Walk-backward”.

The learning curves for both models (Figure A5 and Figure A6) show significant early fluctuations, with training loss spiking before stabilizing and validation loss exhibiting substantial oscillations. However, the accuracy curves steadily improve, reaching peak performance around 0.9 and 0.95 after 40 epochs. This pattern indicates effective learning of complex patterns despite the initial instability in loss, with the second model achieving higher peak accuracy.

### 3.4. Statistical Analysis

We performed the Wilcoxon test using DATAtab [58], based on the 5-fold cross-validation (5CV) results. During each fold of the 5CV, with 75 epochs and early stopping applied, we obtained the validation accuracy for each model and then the test accuracy across all the datasets. The results were combined for each group: Group A includes BiLSTM and ResBiLSTM, Group B includes LSTM and ResLSTM, and Group C includes CNN and ResCNN. We used a significance level of 0.05 for our tests.

The Wilcoxon test results show a *p*-value of 0.215 when comparing Group A, which includes BiLSTM and ResBiLSTM, and Group B, which includes LSTM and ResLSTM. Since the *p*-value is greater than the typical alpha level of 0.05, we fail to reject the null hypothesis, indicating no statistically significant difference between the performance of Group A and Group B.

Additionally, when comparing Group A, which includes BiLSTM and ResBiLSTM, and Group C, which includes CNN and ResCNN, the Wilcoxon test results show a *p*-value of 0.005. This *p*-value is less than the significance level of 0.05, indicating a statistically significant difference between the performance of Group A and Group C.

Our results show that substituting BiLSTM with LSTM and data flipping (Group B) maintains performance, with no statistically significant difference from the original BiLSTM model (Group A). This makes the model lighter while preserving BiLSTM’s capabilities. However, replacing BiLSTM with CNN and data flipping (Group C) results in a significantly lower accuracy, despite also reducing model size. This suggests the LSTM-based approach can effectively approximate BiLSTM’s behavior, while the CNN-based approach lacks the same level of effectiveness, despite both techniques reducing model complexity. The findings address our research questions by demonstrating a viable path to optimize model architecture without sacrificing performance.

### 3.5. Input Sensor Impact

We conducted an analysis to determine the impact of different sensor types, specifically accelerometers and gyroscopes, on the performance of our proposed ResLSTM model. The UCI-HAR dataset was selected for this experiment as it provides comprehensive data from both accelerometers and gyroscopes, enabling a thorough evaluation of their individual and combined contributions to the model’s performance. The WISDM dataset was not utilized due to its limitation to only accelerometer data, which would not allow us to assess the impact of gyroscope data. Similarly, the KU dataset was excluded due to its complexity, and to keep inline with our previous testing on the UCI dataset. The ResLSTM model was trained using the same hyperparameters, which were (0.2,0.2) for the dropout rates, 0.001 for the learning rate, and 32 for the batch size.

The results in Table 8 indicate that the combined use of gyroscope and accelerometer data significantly improves the performance of the ResLSTM model across all evaluated metrics. The model achieved an accuracy of 96.34%, a precision of 96.35%, a recall of 96.33%, and an F1-score of 96.32% when both sensor types were used. This represents a substantial improvement compared to using either sensor type alone. The model’s performance with only accelerometer data was notably better than with only gyroscope data, achieving an accuracy of 82.42% compared to 79.98%, suggesting that accelerometer data may provide more relevant features for the activity recognition task.

The inclusion of both sensor types not only enhances the model’s predictive capabilities but also increases the number of parameters, highlighting a trade-off between model complexity and performance. Despite the increased parameter count, the significant improvement in performance justifies the use of both sensors for this application, this can also suggest that the complementary nature of the data provided by these two types of sensors is crucial for capturing the nuances of human motion, thereby enhancing the model’s ability to accurately classify activities.

### 3.6. Window Size Impact

We also performed an analysis to understand how different window sizes impact the performance of the ResLSTM model. This experiment was conducted using the UCI-HAR dataset, which provides comprehensive data from both accelerometers and gyroscopes. The window sizes tested were 512, 256, 128 (previously used), 64, and 32. The same model architecture and hyperparameters were used for all experiments to ensure a fair comparison, which were (0.2,0.2) for the dropout rates, 0.001 for the learning rate, and 32 for the batch size. See Table 9.

The results indicate that the window size of 128, which was previously used, yielded the highest performance across all evaluated metrics, with an accuracy of 96.34%. The next best window size was 256, with an accuracy of 94.13%. Smaller window sizes, such as 64 and 32, resulted in lower performance, with accuracies of 93.79% and 92.87%, respectively. The largest window size of 512 also showed a slightly lower performance than 256 and 128, with an accuracy of 93.52%.

These findings suggest that a moderate window size of 128 strikes the best balance between capturing sufficient temporal dependencies and maintaining computational efficiency. Larger window sizes, while potentially capturing more context, may introduce noise and increase computational complexity, which does not necessarily translate to better performance. Smaller window sizes, on the other hand, may not capture enough temporal context, leading to reduced model performance.

### 3.7. Comparison with State of the Art Transformer Model

To provide a comprehensive comparison of models within our research, we included an evaluation of the Transformer model alongside our proposed ResLSTM architecture. The goal was to determine how well the Transformer would perform in the context of human activity recognition using the UCI-HAR dataset. For the implementation, Transformer architecture from Keras library [59] was used with a learning rate of 0.001, batch size of 32, activation function of ReLU, and dropout rate of 0.1.

The results (see Table 10) indicate that the Transformer model achieved an accuracy of 91.18%, which did not surpass the performance of our ResLSTM model, which reached an accuracy of 96.34%. This outcome is significant, as it suggests that, for the specific task of human activity recognition with the UCI-HAR dataset, the ResLSTM model provides a superior performance. In terms of precision, recall, and F1-score, the Transformer model also demonstrated strong but slightly lower performance metrics compared to the ResLSTM model. More specifically, the Transformer achieved a precision of 91.20%, recall of 91.17%, and an F1-score of 91.15%, compared to the 96.35%, 96.33%, and 96.32% observed with the ResLSTM model, respectively.

Moreover, it is essential to also highlight the difference in model complexity, particularly the parameter count. The Transformer model, with 7,112,454 parameters, is significantly more complex than the ResLSTM model, which has 576,702 parameters. Despite this increased complexity, the Transformer did not outperform the ResLSTM model, indicating that higher parameter counts and model complexity do not necessarily lead to better performance.

## 4. Discussion

This research aimed to explore whether the ResBiLSTM model in HAR could be replaced by lighter components while maintaining high accuracy, and if the bidirectional nature of BiLSTM could be approximated through data augmentation techniques.

The findings show that the ResLSTM model achieved the highest accuracy of 96.34% in the UCI-HAR dataset, demonstrating its ability to effectively capture complex temporal sequences. This performance is notable, especially when compared to other high-performing models in the literature. In the WISDM dataset, the ResBiLSTM model achieved a higher F1-score of 97.71% compared to the ResLSTM model’s 97.20%, but the ResLSTM model required fewer parameters, indicating a substantial reduction in computational complexity. The minor difference in F1-score is offset by the reduction in parameters, suggesting the ResLSTM with flipped data could be an alternative. For the KU-HAR dataset, the ResBiLSTM model significantly outperformed the ResLSTM model, with an F1-score of 98.72% compared to 96.00%. However, the LSTM model was closer to the ResBiLSTM F1-score at 97.78% with the benefit of lower parameters. This indicates that for datasets with imbalanced class distributions, the bidirectional nature of BiLSTM provides a distinct advantage.

**Figure 15:**
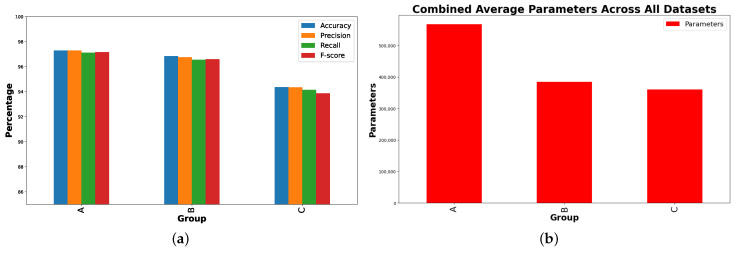
Combined average metrics and parameters across all datasets. (**a**) Average performance metrics. (**b**) Number of parameters.

Across all datasets, Figure 15 shows that the ResBiLSTM model consistently outperformed the ResCNN model in terms of accuracy and F1-score, highlighting the superior temporal processing capability of BiLSTM over CNN, even with flipped data augmentation. The ResCNN model, while showing lower performance, had a significantly lower parameter count, suggesting it is more computationally efficient.

The results indicate that ResLSTM can achieve comparable accuracy with fewer parameters, making it a potential alternative, especially in datasets like UCI-HAR. However, in imbalanced datasets such as KU-HAR and WISDM, ResBiLSTM demonstrated superior performance. The replacement strategies achieved a reduction in model parameters, but the ability to effectively preserve the bidirectional input processing characteristic of BiLSTMs varied across datasets.

The misclassifications between the “Sitting” and “Standing” classes were observed and are primarily due to the similarity in sensor data. The accelerometer and gyroscope readings for these activities often overlap, especially when the person is relatively still in both positions. This similarity makes it challenging for models to distinguish between the two states. Additionally, transitional movements, such as sitting down or standing up, can cause temporary confusion, further complicating accurate classification. Sensor placement and sensitivity also play a crucial role; if the sensors are not positioned optimally or lack the necessary sensitivity, they might fail to capture the subtle differences in posture.

The overfitting was observed in the training of models on the KU-HAR dataset and can be attributed to several factors. A smaller or less diverse dataset, such as KU-HAR, can lead to the model memorizing training samples rather than learning generalizable features, resulting in high training accuracy but poor validation performance. Complex models like ResBiLSTM and ResCNN, with numerous parameters, are particularly prone to overfitting when the dataset does not provide sufficient examples for effective training. The rapid convergence followed by a divergence between training and validation loss in learning curves is indicative of this issue. Furthermore, a lack of robust regularization techniques, such as dropout, L2 regularization, or data augmentation, may have exacerbated the overfitting problem by allowing the model to learn noise in the training data.

This study has several limitations. Its heavy reliance on specific datasets (UCI-HAR, WISDM, KU-HAR) limits the generalizability of the results. Performance might vary significantly with different datasets, potentially limiting the models’ applicability to real-world scenarios. Variability in sensor types, placement, and sampling rates, which the study does not address, could also impact model accuracy and robustness. Moreover, the computational complexity of advanced models like ResBiLSTM and ResCNN might hinder their deployment in real-time or resource-constrained environments, such as wearable devices. The study also focuses on a predefined set of activities, whereas real-world applications often involve a broader and more complex range of activities not covered in this research.

## 5. Conclusions

In conclusion, this study investigated various deep learning architectures for HAR using multiple benchmark datasets (i.e., UCI-HAR, WISDM, KU-HAR). The analysis shows that the Residual Bidirectional LSTM (ResBiLSTM) model consistently achieved the highest accuracy across different datasets, indicating its superior performance and generalization capabilities. The inclusion of residual connections and bidirectional LSTM units effectively enhanced model performance by facilitating efficient learning and reducing misclassifications. The comprehensive evaluation of different models, including BiLSTM, ResBiLSTM, LSTM, ResLSTM, CNN, and ResCNN, provided valuable insights into the strengths and weaknesses of each architecture. The findings highlighted the importance of selecting appropriate models and architectures based on the specific characteristics and requirements of HAR tasks. Despite their high accuracy, the computational complexity of ResBiLSTM and ResCNN models may hinder their real-time deployment in resource-constrained environments. Furthermore, the dependence on specific datasets limits the generalizability of our results, emphasizing the need for further validation across varied and real-world conditions. Future research directions may include exploring hybrid models that combine the strengths of different architectures, incorporating attention mechanisms, and leveraging transfer learning to further enhance HAR performance. Additionally, the integration of multimodal data, such as combining accelerometer and gyroscope data, may offer further improvements in activity recognition accuracy and robustness. By leveraging sensor data and deep learning techniques, this research contributed to the development of advancements that have the potential to enhance a wide range of applications, from healthcare monitoring to fitness tracking and military surveillance, ultimately improving human experiences and decision-making across diverse domains.

## Figures and Tables

**Table 1:** Specific details of the model groups to test.

Group	Model	Hyperparameters Tuned
Group A	No data augmentation with the original ResBiLSTM model	Activation function, learning rate, batch size
	No data augmentation with BiLSTM model	
Group B	Data augmentation with ResLSTM model	Activation function, learning rate, batch size
	Data augmentation with LSTM model	
Group C	Data augmentation with ResCNN model	Activation function, learning rate, kernel size, batch size
	Data augmentation with CNN model	

**Table 2:** Description of the Datasets used for Evaluation.

Dataset Name	No. Sub	No. Ins	No. Classes	Sensors	Activities
UCI-HAR [55]	30	10,299	6	Accel., gyro.	W, WU, WD, S, ST, L.
WISDM [56]	36	1,098,207	6	Accel.	W, J, WU, WD, S, ST.
KU-HAR [57]	90	20,750	18	Accel., gyro.	ST, S, TWS, TWST, SUFS, LD, SUFL, P, JU, PU, SU, W, WB, WC, R, WU, WD, PT.

W = Walking, WU = Walking upstairs, WD = Walking downstairs, S = sitting, ST = Standing, L = Laying, R = Running, JU = jumping, J = Jogging, SUFL= Standing up from laying, SUFS = Standing up from sitting, TWS = Talking while sitting, TWST = Talking while standing, LD = Lying down, SUFL = Standing up from laying down, P = Picking up an object, PU = Pushing up, SU = Sitting up, WB = Walking backward, WC = Walking in a circle, PT = Playing table tennis.

**Table 3:** Specific details of the tested hyperparameters on UCI-HAR dataset for the six models using grid search.

Hyperparameter	Candidate Values
Activation function	(Swish, Relu, Leaky Relu)
Dropout combination	(0.2,0.2) or (0.2,0.3)
Learning rate	(0.01, 0.001, 0.0001)
Kernel size	(3, 5, 7)
Batch size	(32, 64)

**Table 4:** Specific details of the optimal hyperparameters are determined based on the CV procedure applied to the model groups being tested.

Models Name	Activation Function	Dropout Combination	Learning Rate	Batch Size
No data augmentation with the original ResBiLSTM model	Swish	(0.2,0.3)	0.001	32
No data augmentation with BiLSTM model	Leaky Relu	(0.2,0.2)	0.001	64
Data augmentation with ResLSTM model	Relu	(0.2,0.2)	0.001	32
Data augmentation with LSTM model	Relu	(0.2,0.2)	0.001	32
Data augmentation with ResCNN model	Leaky Relu	(0.2,0.3)	0.001	32
Data augmentation with CNN model	Relu	(0.2,0.2)	0.0001	32

**Table 5:** Model results obtained on the UCI-HAR dataset.

Group	Sub. Comp.	*Acc*	*P*	*R*	F1	*Param*	*Time (s)*
A	BiLSTM	95.22	95.72	95.22	95.18	849,534	435.83
	ResBiLSTM	95.96	96.01	95.96	95.96	850,302	414.35
B	LSTM	95.15	95.17	95.15	95.13	576,318	518.39
	ResLSTM	96.34	96.35	96.33	96.32	576,702	295.06
C	CNN	94.2	94.3	94.19	94.19	672,126	405.75
	ResCNN	94.03	94.29	94.03	93.97	408,846	284.95

**Table 6:** Model results obtained on the WISDM dataset.

Group	Sub. Comp.	*Acc*	*P*	*R*	F1	*Param*	*Time (s)*
A	BiLSTM	97.23	97.26	97.23	97.23	283,182	369.36
	ResBiLSTM	97.7	97.74	97.69	97.71	283,438	529.37
B	LSTM	97.14	97.2	97.14	97.16	192,110	368.78
	ResLSTM	97.2	97.21	97.2	97.2	192,238	473.84
C	CNN	96.36	96.52	96.35	96.4	224,046	413.95
	ResCNN	96.42	96.56	96.41	96.45	136,286	308.12

**Table 7:** Model results obtained on the KU-HAR dataset.

Group	Sub. Comp.	*Acc*	*P*	*R*	F1	*Param*	*Time (s)*
A	BiLSTM	98.63	98.42	98.03	98.22	569,462	4507.8
	ResBiLSTM	99	98.83	98.61	98.72	569,974	5487.09
B	LSTM	98.18	97.93	97.69	97.78	385,782	4686.08
	ResLSTM	97.05	96.71	95.8	96	386,038	5235.93
C	CNN	90.59	89.95	89.53	88.12	448,502	2318.02
	ResCNN	94.63	94.49	94.4	94.1	272,982	3083.61

**Table 8:** The performance of the ResLSTM model when trained with data from different sensor types.

ResLSTM Model	Accuracy	Precision	Recall	F1 Score	Parameters
Only Gyroscope	79.98	81.05	79.97	80.07	192,238
Only Accelerometer	82.42	83.24	82.42	83.09	192,238
Gyroscope & Accelerometer	96.34	96.35	96.33	96.32	576,702

**Table 9:** The performance of the ResLSTM model when using different Window Sizes.

Window Size	Accuracy	Precision	Recall	F1 Score	Parameters
512	93.52	93.76	93.51	93.48	576,774
256	94.13	94.23	94.12	94.12	576,726
128 (previously used)	96.34	96.35	96.33	96.32	576,702
64	93.79	93.96	93.79	93.74	576,690
32	92.87	93.17	92.87	92.79	576,684

**Table 10:** Comparison of ResLSTM model with Transformer model on UCI-HAR dataset.

Model	Accuracy	Precision	Recall	F1 Score	Parameters
ResLSTM	96.34	96.35	96.33	96.32	576,702
Transformer	91.18	91.20	91.17	91.15	7,112,454

## Data Availability

Data are contained within the article.

## Appendix A

**Table A1:** Recognition performance of deep learning models on different datasets.

Dataset	Methods	Accuracy
UCI-HAR [55]	CNN, LSTM, BLSTM, MLP, and SVM [17]	92.71%
	GRU, INC, ResNets, CBAM, and attention mechanisms [20]	96.27%
	CNN, GRU, decoder, attention mechanisms, and ResNets [42]	92.4%
	LSTM-CNN [21]	95.8%
	Attention-CNN, CNN and DeepConvLSTM [22]	93.41%
	CNN [10]	97.62%
	multi-input CNN-GRU [11]	96.2%
	1D CNN [12]	95.4%
	1D CNN, ResBiLSTM, and attention [14]	98.37%
	MF CNN [29]	97.32%
	CNN using lego filters [26]	96.27% *
	CNN-LSTM [41]	97.89%
	Transformer [48]	97.2%
Shoaib [60]	CNN, GRU, decoder, attention mechanisms, and ResNets [42]	90%
Pamap2 [61]	CNN, LSTM, BLSTM, MLP, and SVM [17]	91%
	GRU, INC, ResNets, CBAM, and attention mechanisms [20]	90.30% *
	CNN-IMU-2 [18]	93.68%
	Multi-input CNN-GRU [11]	95.27%
	CNN using conditionally parametrized convolution [28]	94.01%
	CNN using lego filters [26]	91.40% *
WISDM [56]	GRU, INC, ResNets, CBAM, and attention mechanisms [20]	99.12% *
	LSTM-CNN [21]	95.85%
	Multi-input CNN-GRU [11]	97.21%
	HARDenseNet [15]	94.65%
	Integration of attention mechanism with multi-head CNN [19]	96.4%
	1D CNN, ResBiLSTM, and attention [14]	99.01%
	MF CNN [29]	97.67%
	CNN using conditionally parametrized convolution [28]	99.6%
	CNN using lego filters [26]	97.51 % *
OPPORTUNITY [62]	GRU, INC, ResNets, CBAM, and attention mechanisms [20]	90.05% *
	LSTM-CNN [21]	92.63%
	CNN [10]	94.2%
	CNN-IMU-2 [18]	87.99%
	CNN using conditionally parametrized convolution [28]	77.31%
	CNN using lego filters [26]	86.01% *
MotionSense [63]	CNN, GRU, decoder, attention mechanisms, and ResNets [42]	92.7%
	MF CNN [29]	98.03%
	Transformer [48]	98.49%
HHAR [64]	CNN, GRU, decoder, attention mechanisms, and ResNets [42]	96.4%
	Transformer [48]	98.72% *
Self-collected dataset [22]	Attention-CNN, CNN and DeepConvLSTM [22]	93.55%
Order picking [65]	CNN-IMU-2 [18]	91.85%
Smartphone dataset [55]	HARDenseNet [15]	91.61%
KU-HAR [57]	1D CNN, ResBiLSTM, and attention [14]	97.89%
	Transformer [46]	99.2%
UNIMIB-SHAR [66]	CNN using conditionally parametrized convolution [28]	77.31%
	CNN using lego filters [26]	74.46% *
RealWorld [67]	Transformer [48]	95.81%
SHL Preview [68,69]	Transformer [48]	94.86%

* F1-Score.
